# Long-term efficiency of pallidal DBS and the role of Levodopa treatment in DYT-GNAL and 18p deletion syndrome associated dystonia: an observational study and review of literature

**DOI:** 10.1007/s00702-026-03109-z

**Published:** 2026-02-03

**Authors:** Johanna Reimer, Friederike Schumann, Katja Lohmann, Thomas G. Riemer, Joachim K. Krauss, Gerd-Helge Schneider, Andrea A. Kühn, Patricia Krause

**Affiliations:** 1 Department of Neurology, Movement Disorder and Neuromodulation Unit, Charité- Universitätsmedizin Berlin, Berlin, Germany; 2 Berlin Institute of Health, Charité-Universitätsmedizin Berlin, Berlin, Germany; 3 Department of Neurology, Park-Klinik Weißensee, Berlin, Germany; 4 Institute of Neurogenetics, University of Lübeck, Lübeck, Germany; 5 Department of Psychiatry and Psychotherapy, Charité-Universitätsmedizin Berlin, Berlin, Germany; 6 Institute of Clinical Pharmacology and Toxicology, Charité-Universitätsmedizin Berlin, Berlin, Germany; 7 Department of Neurosurgery, Hannover Medical School, Hannover, Germany; 8 Department of Neurosurgery, Charité-Universitätsmedizin Berlin, Berlin, Germany; 9 NeuroCure Cluster of Excellence, Charité-Universitätsmedizin Berlin, Berlin, Germany

**Keywords:** DYT-GNAL, DYT25, 18p deletion syndrome associated dystonia, GPi-DBS, Levodopa, Dopa-responsive dystonia

## Abstract

Dystonia due to loss-of-function variants in the *GNAL* gene in DYT-GNAL and 18p-deletion (18pdel) syndrome has been reported to respond well to pallidal deep brain stimulation (GPi-DBS) occasionally, but long-term data is scarce. *GNAL* is implicated in dopamine receptor function, which may explain anecdotal reports of hypokinesia in DYT-GNAL and 18pdel-associated dystonia, a phenomenon that has not yet been systematically reviewed. We retrospectively evaluated a cohort of three patients with *GNAL* variants treated with GPi-DBS, documenting individual long-term outcomes spanning up to 25 years. Dystonia severity was assessed using the Toronto Western Spasmodic Torticollis Rating Scale (TWSTRS) and the Burke − Fahn − Marsden Dystonia Rating Scale (BFMDRS). We also documented bradykinetic symptoms and levodopa response. A literature review was added focusing on DBS outcomes and the effects of levodopa in DYT-GNAL and 18pdel-associated dystonia. In our patients, TWSTRS severity subscale I was reduced by 63% in early (≤ 12months; 20.7 to 7.7 points) and 81% in late follow-up (> 12months; 20.7 to 4 points) after GPi-DBS χ. BFMDRS decreased by 67% and 44%; each with a Kendall’s coefficient of 0.78, indicating a high degree of concordance in improvement trajectories. Two patients exhibited bradykinesia, which was levodopa-responsive in one. GPi-DBS responses have been reported for another ten DYT-GNAL and two 18pdel-patients. Bradykinesia prompted levodopa challenges in 15 patients, resulting in improvement in five. Long-term follow-up data from three DYT-GNAL patients treated with DBS showed sustained improvement in dystonia, particularly in cervical symptoms. Bradykinesia may be an inherent clinical feature of GNAL-related dystonia, warranting further investigation.

**Supplementary Information:**

The online version contains supplementary material available at https://doi.org/10.1007/s00702-026-03109-z.

## Introduction

Pathogenic variants in the G Protein Subunit Alpha L (*GNAL*) gene cause adult-onset primary torsion dystonia (DYT-GNAL). Since its initial identification (Fuchs et al. 2013; Vemula et al. 2013), approximately 100 DYT-GNAL patients have been reported (Lohmann et al. 2025). The inheritance pattern is usually autosomal dominant with high, but not full, penetrance in affected families. Onset typically occurs in early to late adulthood, with a median age at onset (AAO) of 38 years (Lange et al. 2021). In most patients, symptoms begin in the neck and craniocervical region, often progressing to segmental or multifocal involvement, and in some patients, generalized dystonia (https://www.mdsgene.org). *GNAL* is located on the short arm of chromosome 18 (18p11.21). Variable deletions of chromosome 18p result in the 18p deletion (18pdel) syndrome, which is clinically characterized by cognitive impairment, facial dysmorphia, and short stature. Additional features may include spinocerebellar ataxia, facioscapulohumeral muscular dystrophy, and dystonia (Hasi-Zogaj et al. 2015). A deletion of *GNAL* is considered the primary driver of dystonia in 18pdel syndrome (Crosiers et al. 2019).

Despite the increasing recognition of DYT-GNAL, the relationship between genetic variants, clinical expression, and treatment responses, particularly to therapies like deep brain stimulation (DBS), remains to be defined, given the limited number of case reports and therapeutic data.

DBS of the globus pallidus internus (GPi) is an effective treatment option for focal, segmental, and generalized dystonia (Kupsch et al. 2006, Krause et al. 2025). Detailed phenotyping and genetic profiling play an increasingly important role in predicting treatment response, aiming to optimize individual DBS outcomes. Previous reports on DBS effects in DYT-GNAL are mostly limited to single case studies, with the largest series from a single center involving three patients undergoing GPi-DBS (Sarva et al. 2019). In a review of eight published DYT-GNAL GPi-DBS patients, significant improvement of cervical dystonia was observed with mixed responses for limb, laryngeal, and truncal dystonia. Compared to DYT-THAP1 mutation carriers, patients with DYT-TOR1A and DYT-GNAL dystonia demonstrated higher early response rates to DBS, while long-term outcomes were comparable across all three genotypes (Ahn et al. 2019). Additionally, two patients have been reported with DBS treatment in 18pdel-associated dystonia (Vela-Desojo et al. 2019; Duma et al. 2019).

A growing body of evidence implicates the role of dopamine in the pathogenesis of dystonia. The *GNAL* gene encodes the protein Gαolf, which plays a critical role in dopaminergic signal transduction within the striatum. Pathogenic variants in *GNAL* are primarily loss-of-function variants, resulting in reduced Gαolf activity. This leads to an imbalance of the direct and indirect pathways within the basal ganglia, contributing to the pathophysiology of dystonia (Thomsen et al. 2024).

Interestingly, one patient with 18pdel-syndrome with dystonia-parkinsonism and evidence of nigrostriatal degeneration on a dopamine transporter (DaT) scan has been reported. This patient showed partial improvement of bradykinesia and gait disturbances with levodopa treatment (Di Rauso et al. 2024). In our cohort, bradykinesia was observed in two patients, with freezing of gait (FOG) present in one. Although bradykinesia has been described as a stimulation-related side effect in patients treated with chronic GPi-DBS (Huebl et al. 2015; Schrader et al. 2011), in our cohort, symptoms were present prior to DBS treatment. Additionally, the patient reported by Di Rauso et al. (2024) did not receive DBS, suggesting that bradykinesia might be an additional motor feature of *GNAL*-associated dystonia rather than solely a consequence of DBS treatment.

To address this hypothesis, we here systematically evaluate the responses to DBS and medical treatment, including levodopa, alongside a detailed review of motor and non-motor phenotypes in DYT-GNAL and 18pdel-associated dystonia, with a particular focus on bradykinesia. Additionally, we present long-term observational data (up to 25 years) on the effects of bilateral GPi-DBS in three patients with *GNAL* variants treated at our center, contextualizing these findings within the existing literature.

## Methods

The study was approved by the local ethics committee of Charité – Universitätsmedizin Berlin (EA1/120/24) and has therefore been performed in accordance with the ethical standards outlined in the 1964 Declaration of Helsinki and its subsequent amendments. Written informed consent was obtained from all participants. All data supporting the findings of this study are available within the paper and its supplementary information.

*Patients*: We retrospectively analyzed a cohort of three patients with variants in *GNAL* with bilateral GPi-DBS and individual long-term results of up to 25 years of treatment at our center. Patients presented at regular intervals for DBS adjustment and battery check (at least once per year). Video documentation was conducted at baseline and about one year postoperatively, as well as sporadically at later time points, for example, following genetic confirmation or when clinical changes were reported. Dystonia was rated using the Toronto Western Spasmodic Torticollis Rating Scale (TWSTRS) I (severity scale) as well as the Burke-Fahn-Marsden Dystonia Rating Scale (BFMDRS) (movement scale) according to video documentation before DBS (baseline), at an early follow up (FU) time point within the first year of treatment and the longest available postoperative FU (late FU; >12 months). TWSTRS II (disability subscale) and III (pain subscale) scores, as well as Unified Parkinson’s Disease Rating scale (UPDRS) motor examination (part III), could not be assessed retrospectively but were sporadically assessed during clinical routine follow-ups. Furthermore, we evaluated bradykinetic symptoms, DaT scan results, and levodopa response wherever data were available, including a review of clinical records and video documentation.

*Genetics*: The genetic variants were detected either in a research setting (DYSTRACT consortium) through microarray analyses (unpublished data, Patient 1 and 2) or by diagnostic testing using gene panel analysis (Patient 3).

*Review of literature*: Data and publications included in our literature review were collected and analyzed up to March 31st, 2025. The PubMed database was searched for literature on DYT-GNAL- and 18pdel-associated dystonia with the focus on published cases with given information on additional phenotyping and/or applied therapies. Use of the key words “GNAL dystonia”, “DYT 25”, “DYT GNAL” and “18p syndrome AND dystonia” provided 115 results for DYT-GNAL and seven results for 18pdel-syndrome-associated dystonia. We retrieved 21 DYT-GNAL patients fulfilling the criteria on phenotypical description (written/disease-specific motor scales or video documentation) and/or given information on applied therapies, as well as 19 patients with 18pdel-associated dystonia.

*Statistics*: Statistical analysis of age, disease duration, and dystonia scores was conducted using SPSS (IBM Corp. Released 2023. IBM SPSS Statistics for Windows, Version 29.0.2.0 Armonk, NY: IBM Corp). Data are given as mean ± standard deviation (SD) according to Cohen’s unless specified otherwise. Changes from baseline TWSTRS I and BFMDRS motor subscale (pre-DBS) at early and late follow-up were analyzed using non-parametric Friedman test for repeated measures. A p-value < 0.05was considered to be significant. Kendall’s coefficient (W) was used to quantify the effect size and calculated using the equation W = X^2^/(k*[n-1]) (X^2^ = Friedman test statistic, k = number of patients, n = number of timepoints). Values between 0.6 and 0.8 are generally interpreted as a strong level of agreement indicating consistent patterns of change over patients, whereas values above 0.8 indicate a very strong effect.

## Results

### Patient cohort from our center

*Demographics*: Patients (video documentation available in the Supplementary Online Resource Videos 1, 2, 3) treated at our center (1 female) had a mean AAO of 31.7 years (±3.3 ; range 28 –36 years) and a mean age at DBS surgery of 37 years (±3.6 ; range 34–42 years). Detailed case vignettes are provided below. Additional information on genetic variants can be found in Table 1.

**Table 1:** Demographic overview, phenotypical characterization, genetic findings, and comorbidities of the DYT-GNAL patients treated with GPi-DBS in our center

	Patient 1 L-7947	Patient 2 L-11515	Patient 3 NA
Age at onset	36	31	28
Sex	Male	Female	Male
Side onset	Neck	Neck, dystonic tremor	Neck
Distribution	Generalized	Generalized	Segmental
Clinical phenotype	Torticollis, blepharospasm, oromandibular dystonia, trunk, right arm, gait, pain	Neck, dystonic tremor, trunk, right upper and lower limb dystonia, pain	Torticollis, trunk, dystonic tremor right limb, dysarthria, pain
Comorbidities	Depression (BDI-II 31)	COPD, migraine headaches	None
Family history	Positive	Positive	Negative
Genetic testing	DYSTRACT study	DYSTRACT study	diagnostic genetic testing (CeGaT)
Method	Gene panel analysis, research-based microarray analysis	Exome sequencing, gene panel analysis, research-based microarray (GSA) analysis	Commercial gene panel analysis
Genetic variant	het. 1.3 Mb-deletion, incl. *GNAL*	het. 1.3 Mb-deletion, incl. *GNAL*	het. canonical splice site variant
	(PennCNV: chr18:10581615–11902127, hg19; deleted genes: *CHMP1B; GNAL; MPPE1; PIEZO2; SLC35G4*)	(PennCNV: chr18:10597874–11902127, hg19; deleted genes: *CHMP1B; GNAL; MPPE1; PIEZO2; SLC35G4*)	c.504 + 1G > T; p.? (NM_182978.4)
In-silico prediction	CADD-SV: 38	CADD-SV: 38	SpliceAI: 1.0 (100% probability) for donor loss CADD score: 34
ACMG	PVS1, PM2, PP3 → pathogenic	PVS1, PM2, PP3 →pathogenic	PVS1, PM2, PP3 → pathogenic

**Abbreviations** *BDI*  Beck depression inventory, *COPD*  chronic obstructive pulmonary disease, *CeGaT*  Center for genomics and Transcriptions, *CNV*  copy number variation, *chr*  chromosome, *het.* heterozygous, *CADD-SV* Combined annotation dependent depletion for structural Variants, *ACMG*  American college of medical genetics and genomics (Richards et al. 2015), *PVS1* Pathogenic very strong 1, *PM2*  Pathogenic moderate meaning absent from controls (or at extremely low frequency if recessive), *PP3* multiple lines of computational evidence support a deleterious effect on the gene or gene product

Age at onset (AAO) is given in years. Regarding patient 1 and 2, the provided breakpoints are based on the microarray analysis.

*Patient 1*: This male patient, with a positive family history for dystonia, developed spasmodic torticollis at the age of 36. Genetic testing revealed a 1.3 Mb deletion including the *GNAL* gene (DYSTRACT, unpublished data) in both the patient and his affected father (who presented with cervical dystonia and parkinsonism). Treatment with trihexyphenidyl, clonazepam, baclofen and cervical botulinum toxin injections provided only partial and transient benefit, leading to evaluation for bilateral GPi-DBS at the age of 44 years. Preoperatively, TWSTRS I was 23/35 points and BFMDRS motor score 27.5/120 points. In addition to dystonic features, both patient and father presented with bradykinesia and slight freezing of gait (FOG). The patient´s DaT-Scan was unremarkable, but a standardized levodopa challenge prior to DBS markedly improved bradykinesia and FOG, with UPDRS III decreased by 61% (from 26/132 points to 10/132 points). Oral medication and botulinum toxin injections were discontinued postoperatively due to the sufficient DBS effect. After initial improvement during the first year, secondary generalization of dystonia developed, affecting the trunk, left upper limb, and both legs. Adjustment of stimulation parameters improved cervical and truncal dystonia and gait (TWSTRS I DBS-OFF 20/35; DBS-ON 6/35, BFMDRS DBS-ON 9/120). Eleven years post implantation, benefit for cervical dystonia remained stable, though refractory painful dystonia of the left upper limb persisted. Transient stimulation-induced side effects included transient dysarthria, paresthesia, and gait disturbance. Under 300 mg/day of levodopa, the patient reported a smoother gait and increased walking speed. Gait and bradykinesia worsened during (short-term) DBS-OFF, while higher stimulation frequencies additionally increased FOG. Long-term DBS-OFF was not tolerated due to exacerbating neck and back pain. After 11 years, TWSTRS I remained improved (8/35). Botulinum toxin treatment for the upper left limb was reintroduced 13 years post-surgery due to progressive dystonia and pain (BFMDRS 16/120).

*Patient 2*: This female patient developed cervical dystonia at age 31 years with secondary generalization to the face and limbs and intermittent phasic head movements. Family history was possibly positive (sister with head tremor; no genetic testing). The patient´s genetic testing revealed a heterozygous deletion of the entire *GNAL* gene (DYSTRACT, unpublished data). Botulinum toxin injections and trihexyphenidyl had minimal effect; they provided partial benefit (phasic movements). Preoperative TWSTRS I was 24/35 and BFMDRS was 26/120. Combined GPi and Nucleus ventralis intermedius of the thalamus (VIM)-DBS was performed at the age of 34 years, given the prominent phasic movements. Postoperatively, TWSTRS I and BFMDRS improved to 6/35 and 5/120, respectively. The patient reported subjective pain relief, but systematic documentation (TWSTRS III) was not available. At the age of 45 years, revision was required due to pressure necrosis and wound healing deficit. Due to infection, both implantable pulse generators (IPG) were removed. During this temporary DBS-OFF phase, BFMDRS was 12/120 points under oral tetrabenazine (up to 150 mg/d) treatment. After antibiotic therapy, GPi stimulation was resumed, while VIM stimulation was discontinued. Tetrabenazine again improved phasic movements but was later stopped when symptoms resolved under GPi stimulation alone. After 25 years of bipallidal DBS, TWSTRS I was 3/35 and BFMDRS 5/120. Side effects included mild dysarthria and dysphagia. Pain relief remained stable, and the only sign of possible bradykinesia was a mildly increased turning step count, which was not bothersome. DaT-Scan and levodopa challenge were therefore not pursued.

*Patient 3*: This male patient developed jerky head movements at the age of 28 years and cervical dystonia within two years, accompanied by severe neck and back pain by age 33 years. MRI showed bilateral pallidal T2* hypointensities suggesting iron accumulation, while copper metabolism was normal. Family history was negative. Diagnostic genetic testing identified a heterozygous *GNAL* splice variant (c.504 + 1G > T; p.?) classified as pathogenic. Botulinum toxin injections were ineffective and poorly tolerated. Anticholinergics were discontinued due to side effects. Preoperatively, TWSTRS I was 13/35, TWSTRS II (disability) 7/30, TWSTRS III (pain) 6/20, and BFMDRS 6/120. GPi-DBS was implanted at age 35. After the first 6 months with limited improvement (TWSTRS I 11/35, TWSTRS II 11/30, TWSTRS III 5/20, BFMDRS 2/120), optimization of stimulation led to near-complete relief of cervical dystonia and pain, sustained at 9 years postoperatively (TWSTRS I 1/35, TWSTRS II 0/30, TWSTRS III 0/20). A mild increase in upper limb dystonia developed over time with some functional impairment (BFMDRS 6/120). Slight pre-existing bradykinesia (arrhythmic alternating movements present in preoperative video documentation and postoperative follow-up evaluations) was not bothersome, and levodopa challenge was deemed unnecessary.

*DBS effects*: Changes of motor ratings, including TWSTRS I and BFMDRS (sub)scales, were analyzed at two timepoints: early follow-up (mean 25.3 ±18.8 weeks; range 2–48 weeks) and late follow-up (mean 15 ±7.1 years; range 9–25 years). TWSTRS I improved from 20.7/35 ± 5.4 points at baseline to 7.7/35 ± 2.4 points at early follow-up and to 4/35 ± 2.9 at late follow-up. A Friedman test revealed a Kendall’s coefficient (W) of 0.78. No significant difference in TWSTRS I scores across the three time points regarding the p-value was reached (χ²(2) = 4.667, *p* = 0.097). The BFMDRS motor subscale improved from 16/120 ± 8.2 at baseline to 5.3/120 ± 2.9 at early follow-up and 9 /120 ± 5 at late follow-up. A Friedman test showed a W of 0.78 (χ²(2) = 4.667, *p* = 0.097).

**Fig. 1:**
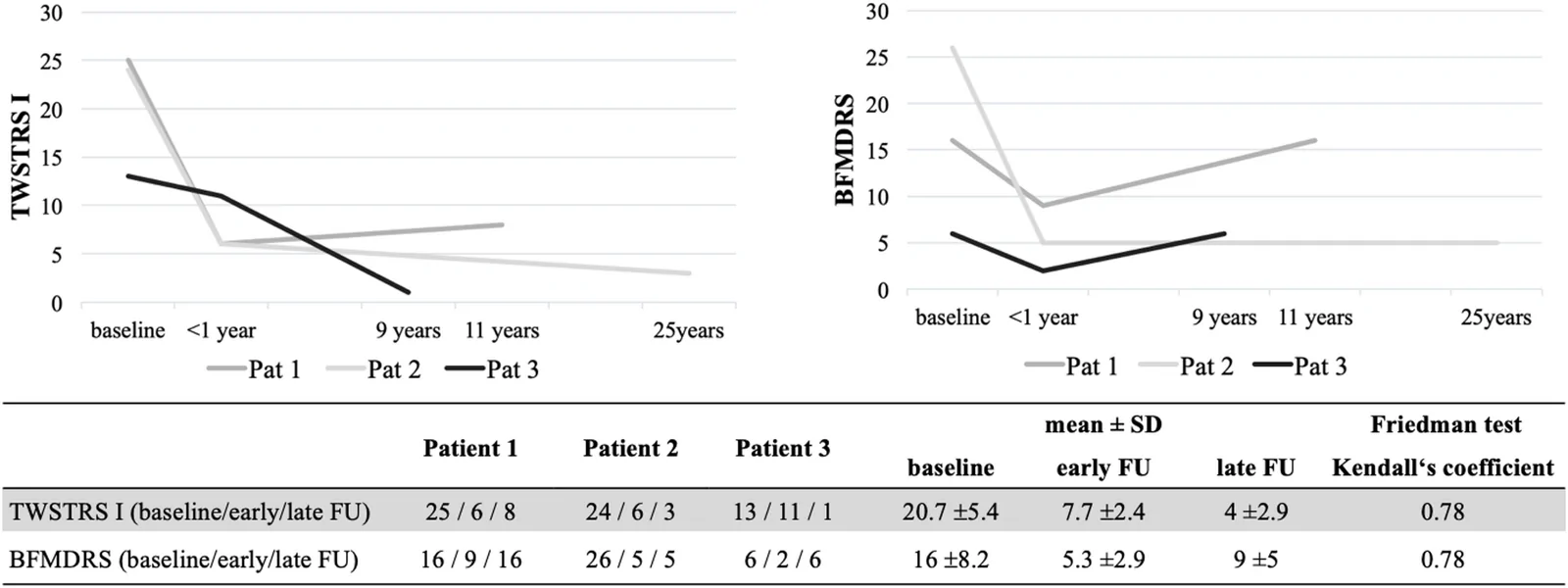
specific dystonia motor rating scales of the DYT-GNAL patients 1–3treated with bipallidal deep brain stimulation (DBS) in our center at baseline (pre DBS), early follow up (FU) (< 12 months) in DBS-ON and late FU (> 12 months, range 9–25 years) in DBS-ON; means given with standard deviation (SD) and Kendall’s coefficient (W) calculated from Friedman test statistic across all three timepoints; a W of 0.78 is interpreted as a strong level of agreement indicating consistent patterns of change over patients. **Abbreviations** *FU* follow up, *DBS* deep brain stimulation, *TWSTRS * Toronto Western Spasmodic Torticollis Rating Scale I (severity scale), *BFMDRS* Burke-Fahn-Marsden Dystonia Rating Scale (movement scale)

Symptom progression of dystonia was observed in Patient 1 (generalization to upper and lower limbs within the first eleven months of DBS treatment) and Patient 3 (worsening of upper limb dystonia after 8 years of DBS treatment), despite continuous DBS therapy (Fig. 1).

### Review of literature

Along with data from our three patients, we present and review clinical information, treatments, and therapeutic responses from a total of 24 DYT-GNAL and 19 18pdel-associated dystonia patients. Each patient was assigned an ID (1–43). Individual treatment responses are detailed in Table 2, while genetic variants and additional phenotype information for all patients can be found in the in Supplementary Online resource 4.

**Table 2:** Individual treatment responses of reviewed DYT-GNAL and 18pdel-associated dystonia patients with given information on applied therapies (Patient ID 4–41), for treatment responses of patients 1–3 see Table 1, in case no information on applied therapies is given, patient IDs are not listed in this table

Patient ID	4	5	6	7	9	11	12	13	14	15	17	18	19	20	22	23	24	25	26	27	28	30	31	32	33	34	35	37	41
	Magistrelli 2024	Racki 2023	Romito 2023	Fan 2022	Kumar 2019	Ahn 2019	Pandey 2019	Sarva 2019	Sarva 2019	Sarva 2019	Dos Santos 2016	Carrechio 2015	Carrechio 2015	Carrechio 2015	Dobrici 2014	Ziegan 2014	Ziegan 2014	Braccia 2024	Braccia 2024	Rauso 2024	Vela Desojo 2019	Duma 2019	Crosiers 2018	Kumar 2017	Esposito 2014	Kowarik 2011	Postma 2009	Nasir 2006	Awaad 1999
DBS		++	++			++	++	++	++	++		++	++			++					++	++							
Botulinum toxin	+	−	−	+	+	−	++	?	++	?	++	+			+	−	++	++			−		++				++	++	
Trihexyphenidyl	+					−	−	?													−	+					+		+
Benzodiazepines			−	+				+													−	+			−	−			+
Muscle relaxants						−	−	+	?												−								++
Levodopa	−					−	+					−		−			−		+	+	−			+	−	−	−		−
Gabapentinoid			−					+														+							
Tetrabenazin								?				−																	
Profenamin										?																			
Propranolol								?																					
Biperiden																									−	−			
Topiramat								?																	++				
Tiaprid																										−			

**Symbols** (++) = sufficient response; (+) = some response; (-) = no response; (?) = therapy applied but no outcome reported; no entry means that there was no mentioning on whether or not the treatment was applied

*Clinical presentation*: In the DYT-GNAL subgroup (54% female), the mean AAO was 31 ± 15 years. The cervical region was the predominant site of dystonic symptoms, with generalized dystonia occurring in 36% of cases. Additional motor symptoms consistent with hypokinetic features including bradykinesia (*n* = 9), camptocormia (*n* = 3), and freezing of gait (FOG) (*n* = 1). Other motor symptoms, such as myoclonus (*n* = 3), resting tremor (*n* = 1), and ataxia (*n* = 1), were occasionally reported.

In the 18pdel-associated dystonia cohort (72% female), the mean AAO was 27.5 ± 12.7 years, with specific AAO data not given in five patients. Generalized dystonia was reported in 47% of cases. Hypokinetic motor symptoms included bradykinesia (*n* = 4), rigidity (*n* = 2), FOG (*n* = 1), and camptocormia (*n* = 1). Other motor features reported were blepharospasm (*n* = 4), myoclonus (*n* = 4), ataxia (*n* = 1), chorea (*n* = 1), and pyramidal tract signs (*n* = 2).

*Imaging*: Two DYT-GNAL patients (patient IDs: 1 [present study], and 20 (Carecchio et al. 2016) underwent a DaT scan, with patient 20 showing signs of nigrostriatal neurodegeneration. Two 18pdel-associated dystonia patients (patient IDs: 26 (Braccia et al. 2024), 27 (Di Rauso et al. 2024)) also received a DaT scan, with Patient 27 showing moderate reduction in presynaptic dopaminergic uptake in the left caudate nucleus and both putamina.

*Deep brain stimulation*: In addition to our three DYT-GNAL patients, we identified ten DYT-GNAL (patient IDs: 5 (Racki et al. 2023), 6 (Romito et al. 2023), 11 (Ahn et al. 2019), 12 (Pandey et al. 2019), 13, 14, 15 (Sarva et al. 2019), 18, 19 (Carecchio et al. 2016), 23 [Ziegan et al. 2014] and two 18pdel-associated dystonia patients (patient IDs 28 (Vela-Desojo et al. 2019), 30 (Duma et al. 2019)) treated with GPi-DBS from the literature. AAO for all 15 was 31.6 ± 12.7 years, with a disease duration from symptom onset to DBS of 11.9 ± 8.3 years. Stimulation settings were available for 12 patients. The stimulation amplitude ranged from 2 to 6 V (mean 2.8 ± 1.0 V; in two patients amplitude was administered in mA, range 2-5.1 mA, mean 3.65 ± 1 mA). The stimulation frequency ranged from 125 to 210 Hz (mean 163 ± 28 Hz), and the stimulation width ranged from 60 to 150 µs (mean 98 ± 27 µs).

Long-term follow-up (mean FU duration 5.9 ± 6.2 years, range 2 weeks to 25 years) showed sustained reduction of cervical dystonia. Limb dystonia responded well in seven patients (patient IDs: 2, 11, 12, 13, 15, 25, 27 ). Laryngeal dystonia showed improvement in patients 19 and 30. Five patients reported neck pain at baseline (patient IDs: 1, 2, 3 13, 14 ), with pain reduction observed in four after DBS. Symptom-specific scales, such as TWSTRS I and BFMDRS for motor symptoms, and TWSTRS III for pain, were reported inconsistently. Therefore, statistical analysis over the reviewed cohort was not possible. Progression of dystonia despite chronic neuromodulation occurred in three cases (patient IDs: 1, 3, 15), with limited effects on trunk and limb symptoms. Writer’s cramp did not improve in one patients (patient ID: 13). Dysarthria persisted or did not fully respond in two patients (patient IDs: 6, 15 ) and occurred as a stimulation associated side effect in patient 2. Laryngeal dystonia did not respond to DBS in Patient 14 and occurred as a DBS side effect in Patient 2. For individual treatment responses, side effects, and DBS settings, see Supplementary Online resource 5

*Levodopa response*: A levodopa challenge was done in seven DYT-GNAL patients (patient IDs: 1, [present study], 4 (Magistrelli et al. 2024), 11 (Ahn et al. 2019), 12 (Pandey et al. 2019), 18, 20 (Carecchio et al. 2016), 24 (Ziegan et al. 2014)). Patient 1 demonstrated a significant 61% reduction in motor symptoms in the motor part of the Unified Parkinson’s Disease Rating Scale (UPDRS III) (26 points off-medication vs. 10 points on-medication), along with improved gait and reduced clumsiness with a daily levodopa dose of 300 mg and prior to DBS. Dystonia remained unaffected. FOG persisted under GPi-DBS treatment. Patient 12 reported partial reduction of camptocormia with 400 mg levodopa per day. For the other five patients no beneficial effects of dopaminergic treatment have been reported.

A levodopa challenge was conducted in eight 18pdel-associated dystonia patients (patient IDs: 26 (Braccia et al. 2024), 27 (Di Rauso et al. 2024)) 28 (Vela-Desojo et al. 2019), 32 (Kumar et al. 2017], 33 (Esposito et al. 2014), 34, 35, 41 (Crosiers et al. 2019)). Patient 27 showed a 24% reduction in the UPDRS III (25 points off-medication vs. 19 points on-medication), accompanied by improvements in gait and coordination under a daily levodopa dose of 300 mg, while dystonic symptoms remained unchanged. Patient 26 reported moderate benefit for limb hypokinesia and clumsiness. Patient 32 showed improved lower limb dystonia and gait after 6 months of 300 mg levodopa per day. No positive effects were noted in the remaining five patients.

## Discussion

In this study, we report long-term follow-up data on three dystonia patients with pathogenic *GNAL* variants treated with GPi-DBS at our center and contextualize these findings with the existing literature on pharmacological management and DBS outcomes in ten DYT-GNAL and two 18pdel-associated dystonia. Given our observations of bradykinesia and freezing of gait (FOG) in DYT-GNAL patients, we also present a systematic evaluation of pharmacological treatment, including levodopa, alongside a comprehensive review of additional motor and non-motor phenotypes in both dystonia subtypes. To our knowledge, this is the first study to systematically assess levodopa responsiveness and bradykinetic symptoms in DYT-GNAL and 18pdel-associated dystonia.

### DBS effects in DYT-GNAL and 18pdel-associated dystonia

Across all 15 patients with *GNAL* variants and 18pdel-associated dystonia treated with bilateral pallidal DBS, sustained improvement in cervical dystonia was observed over follow-up periods of up to 25 years with comparable patterns of improvement across our three patients indicated by a high Kendall’s coefficient of concordance (W = 0.78). While cervical dystonia consistently showed a good response to DBS, outcomes for limb dystonia and speech were more variable. Furthermore, (secondary) generalization is common in the analyzed DBS cohort with only two patients with reportedly focal distribution (patient IDs: 14 (Sarva et al. 2019), 18 (Carecchio et al. 2016)). However, due to the lack of standardized motor scores such as TWSTRS I and BFMDRS, for most reviewed patients, no formal analysis could be performed for the DBS cohort.

In the reviewed cohort, including our three patients, AAO did not differ significantly between DYT-GNAL and 18pdel-associated dystonia patients who did or did not receive DBS. Pain, present in all three of our patients and mentioned in Patients 13 and 14 (Sarva et al. 2019), may be an important factor to pursue DBS. Available patient histories indicated a relevant reduction in pain; however, symptom-specific scores, such as TWSTRS III for pain quantification, were unavailable for most reviewed patients and for our patients.

### Bradykinesia and Levodopa treatment in DYT-GNAL and 18pdel-associated dystonia

All reviewed patients received guideline-based medical treatment (AWMF 2021). Botulinum toxin injections were highly effective for cervical dystonia, orofacial dystonia, and limb dystonia (compare Table 2).

Along with our own observation of bradykinesia and FOG in one of our DYT-GNAL patients, Parkinson’s Disease (PD)-like symptoms in both DYT-GNAL and 18pdel-associated dystonia prompted a levodopa challenge in 15 patients, with some improvement seen in five. Only one 18pdel-associated dystonia patient showed improvement in dystonia itself.

Bradykinesia and FOG have been reported as potential stimulation-associated side effects of chronic bipallidal neurostimulation (Huebl et al. 2015; Schrader et al. 2011). Since one or both symptoms were evident in preoperative video recordings of our patient and persisted during the stimulation off-state, we do not consider these symptoms to be stimulation-induced. We can only speculate whether patients with DYT-GNAL may be more susceptible to a worsening of preexisting bradykinesia under chronic neurostimulation of the GPi. Since most published patients did not receive GPi-DBS, we interpret these PD-like features as a potential additional motor manifestation of DYT-GNAL dystonia, rather than solely being a DBS side effect.

The functional impairment of Gαolf due to pathogenic *GNAL* variants and deletions may underlie the observed dopaminergic dysregulation. Gαolf plays a crucial role in striatal signaling by directly coupling dopamine 1 (D1R) and adenosine type 2 (A2A) receptors to adenylyl cyclase, leading to an activation of the cAMP pathway (Corvol et al. 2001). Simultaneously, Gαolf interacts with dopamine 2 receptors (D2R), inhibiting adenylyl cyclase and modulating dopaminergic signaling through the indirect pathway (El Atiallah et al. 2024). Oral levodopa intake may increase presynaptic dopamine release, compensating for the decreased responsiveness of D1R and D2R by increasing ligand concentration and receptor saturation, thereby maximizing residual receptor activity. Furthermore, levodopa modulates cholinergic and glutamatergic neurons in both the direct and indirect pathways, which are essential for motor control (De Deurwaerdere et al. 2017).

As previously noted, levodopa-response was observed in only one-third of patients. We hypothesize that variations in the response to dopaminergic therapy might differ due to varying residual function of the D1R and D2R.

PD-like symptoms led to DaT scans in four patients, which were normal despite clinically relevant bradykinesia and FOG in two patients. Only one DYT-GNAL patient showed DaT scan results suggestive of nigrostriatal degeneration, but the tremor in this patient was not levodopa-responsive (Carecchio et al. 2016). Given the patient´s documented traumatic brain hemorrhage, it cannot be stated with diagnostic certainty that the DaT scan findings indicate primary neurodegeneration, as they may instead reflect secondary injury to the basal ganglia resulting from the trauma. Di Rauso et al. reported a pathological DaT scan in a 56-year-old patient with dystonia-parkinsonism and 18pdel-syndrome, who responded to levodopa with improvement of bradykinesia, rigidity, and gait. Given the typical AAO, coexisting neurodegenerative PD cannot be excluded (2024). Resting tremor, accordingly, is not a common symptom of DYT-GNAL dystonia according to MDSGene (Lange et al. 2021).

Considering the receptor-level dysfunction in D1R and D2R, and the resulting pathway imbalance, pathological findings on DaT scans are not necessarily expected. Thus, normal, or nonspecific DaT scan results align with the underlying pathophysiology and do not contradict our clinical observations. In DYT-GNAL patients with PD-like symptoms, a levodopa trial should be considered. Instrumental diagnostics may benefit from focusing less on dopamine transporter density (e.g. DaT scans) and more on receptor function. Future studies exploring the potential of ¹²³I-iodobenzamide (IBZM) single-photon emission computed tomography (SPECT) as a suitable alternative diagnostic tool would be of particular interest.

### Genetics in DYT-GNAL and the reviewed 18pdel-dystonia cohort

In our patients, the loss-of-function effect is evident in the whole gene deletions observed in Patients 1 and 2, as well as in Patient 3 with a splice site variant. To our knowledge, the variant (c.504 + 1G > T; p.?) in Patient 3 has not been previously reported. It is classified as pathogenic according to ACMG criteria (Richards et al. 2015).

A heterozygous deletion of *GNAL* is considered the primary cause of dystonia in 18pdel-syndrome with dystonic features. While other genes are also deleted in this chromosomal region, none of them have been directly associated with dystonia, though they may contribute to additional motor and non-motor features. For instance, the *LAMA1* gene, located close to *GNAL*, is involved in cerebellar development and may play a role in symptoms such as ataxia and myoclonus (Crosiers et al. 2019). Additionally, it was speculated that the positive response to tiapride in Patient 34 may be linked to the *DLGAP1* gene deletion, which plays a key role in glutamatergic neurotransmission (Esposito et al. 2014). Clinically, we observed overlapping dystonic phenotypes between DYT-GNAL and 18pdel-associated dystonia. However, given the presence of additional gene deletions in 18pdel-syndrome, we believe it is important to evaluate these conditions separately.

### Strengths and limitations of the study

This study provides a comprehensive phenotypic and treatment-related characterization of two rare genetic forms of dystonia, DYT-GNAL and 18pdel-associated dystonia, through detailed clinical observation and structured treatment evaluation. It offers an in-depth analysis of medical treatment regimens and their effects, including botulinum toxin and levodopa. Notably, we present long-term follow-up data of GPi-DBS for up to 25 years in DYT-GNAL dystonia, reporting outcomes from three patients being regularly treated in our center. In addition, we describe a novel observation of PD-like features, such as bradykinesia and FOG, in DYT-GNAL dystonia patients, with preliminary evidence suggesting potential levodopa responsiveness in at least a subset of patients. These findings may expand clinical understanding and management of *GNAL*-related movement disorders.

Due to the retrospective design and the nature of this study as a review of multiple case reports on a rare genetic form of dystonia, the dataset lacks consistency and homogeneity, particularly in terms of TWSTRS I, II and III scores and the BFMDRS motor and disability scales. Additionally, the small sample size in our cohort limits statistical analysis of motor scales to non-parametric tests. Kendall’s coefficient of concordance (W) was used to assess the consistency and extent of change across patients, since it is less dependent on sample size compared to p-value based significance testing. Furthermore, the reviewed sample is highly heterogeneous in terms of age at onset, age at surgery, genetic variants, and dystonia distribution, making it challenging to generalize these results to other cohorts.

## Conclusions

Genetic testing of dystonia patients is increasingly important due to growing evidence of variable outcomes following DBS. Combining our own cases with those from the literature, the results support the use of DBS as a safe and long-lasting treatment option for medically refractory DYT-GNAL dystonia patients, particularly for cervical dystonia. However, the suboptimal effects of DBS on truncal and limb dystonia should be addressed during individual counseling to set realistic expectations for treatment outcomes in DYT-GNAL and 18pdel-associated dystonia.

Although DYT-GNAL dystonia is generally considered non-responsive to levodopa, PD-like features were commonly observed in both DYT-GNAL and 18pdel-associated dystonia, with five patients reporting symptom improvement after levodopa administration. While comprehensive descriptions of clinical phenotypes and individual disease trajectories are still limited, they are essential to determine whether bradykinesia is a feature of DYT-GNAL dystonia or specific *GNAL* variants. Such findings could help reclassify DYT-GNAL dystonia as a combined rather than isolated form of dystonia. We recommend screening for PD-like signs pre- and postoperatively and performing a levodopa challenge to assess potential therapeutic benefit.

## Supplementary Information

Below is the link to the electronic supplementary material.

Supplementary Material 1 Video 1: Video documentation of the motor examination for Patient 1. Video timepoints given as (minute:second). Performed before deep brain stimulation (DBS) (0:00), after one year in DBS-ON (1:20), after three years in DBS-OFF (1:56) and DBS-ON (3:14) and eleven years after surgery in DBS-OFF (4:42) and DBS-ON (210Hz, 60µs) (6:35) as well as DBS-ON (210Hz, 60µs) +300mg levodopa/day (8:52)

Supplementary Material 2 Video 2: Video documentation of the motor examination of Patient 2. Video timepoints given as (minute:second). Before deep brain stimulation (DBS) (0:00), one month after DBS in DBS-ON (internal globus pallidus: 80Hz, 210µs) (1:35), as well as 25 years post-surgery in DBS-ON (internal globus pallidus: 185Hz, 90µs) (3:36)

Supplementary Material 3 Video 3: Video documentation of the motor examination of Patient 3. Video timepoints given as (minute:second). Before deep brain stimulation (DBS) (0:00) and six years post-surgery in the DBS-ON (179Hz, 120µs) (2:38)

Supplementary Material 4

Supplementary Material 5
