# Supplementary material for: Long-term efficiency of pallidal DBS and the role of Levodopa treatment in DYT-GNAL and 18p deletion syndrome associated dystonia: an observational study and review of literature
Source: J Neural Transm (Vienna). 2026 Feb 3;133(9):2907–16. doi: 10.1007/s00702-026-03109-z (PMC13687376; doi:10.1007/s00702-026-03109-z)
Supplement: Supplementary file 1 [file 702_2026_3109_MOESM4_ESM.docx]

**Supplementary Information (SI) – ESM 4**

**Long-term efficiency of pallidal DBS and the role of Levodopa treatment in DYT-GNAL and 18p deletion syndrome associated dystonia: An observational study and review of literature**

**Journal of Neural Transmission**

**Authors:** Reimer, Johanna^1,2^; Schumann, Friederike^1,3^; Lohmann, Katja^4^; Riemer, Thomas G.^5^; Krauss, Joachim K.^6^; Schneider Gerd-Helge^7^; Kühn, Andrea A.^1,8^; Krause, Patricia^1^

1 Department of Neurology, Movement Disorder and Neuromodulation Unit, Charité-Universitätsmedizin Berlin, Berlin, Germany

2 Berlin Institute of Health, Charité-Universitätsmedizin Berlin, Berlin, Germany

3 Department of Neurology, Park-Klinik Weißensee, Berlin, Germany

4 Institute of Neurogenetics, University of Lübeck, German

5 Department of Psychiatry and Psychotherapy, Charité-Universitätsmedizin Berlin, Berlin, Germany; Institute of Clinical Pharmacology and Toxicology, Charité-Universitätsmedizin Berlin, Berlin, Germany

6 Department of Neurosurgery, Hannover Medical School, Hannover, Germany.

7 Department of Neurosurgery, Charité-Universitätsmedizin Berlin, Berlin, Germany

8 NeuroCure Cluster of Excellence, Charité-Universitätsmedizin Berlin, Berlin, Germany

**Corresponding author**: Patricia Krause, MD, patricia.krause@charite.de; ORCID 0000-0001-9063-0556

| ID | literature | sex | AAO | Mutation | Family  history | Site | F | L | C | T | UL | LL | T | parkinsonian symptoms | DaT scan | Levodopa | Additional features | DBS |
| --- | --- | --- | --- | --- | --- | --- | --- | --- | --- | --- | --- | --- | --- | --- | --- | --- | --- | --- |
| 1 | present study | m | 36 | 1.3 Mbp deletion including GNAL | + | G | x |  | x | x | x | x |  | bradykinesia and FOG | yes | 300mg/d, levodopa challenge off-med UPDRS III= 26 points vs. on-med UPDRS III= 10 points; improvement of 61%), no benefit for dystonia | depression | yes |
| 2 | present study | f | 31 | 1.3 Mbp deletion including GNAL | + | G | x | x | x | x | x | x |  | no | no | no | COPD, migraine headaches | yes |
| 3 | present study | m | 28 | GNAL heterozygous variant c.504+1G>T; p.? | - | S |  |  | x |  | x |  | x | slight upper limb bradykinesia | no | no | mild cognitive impairment | yes |
| 4 | Magistrelli 2024 | m | 15 | GNAL compound heterozygous c.1163‐1G>A and c.899A>T (p.Gln300Leu) | - | G | x |  | x | x | x |  |  | upper limb bradykinesia |  | 300mg/d, no effect | dysmorphic face, sensoryneuroal hearing loss, moderate intellectual disability | no |
| 5 | Racki 2023 | f | 7 | GNAL c.394A > G: p.Lys132Glu | NA | S | x |  | x | x | x |  | x | NA | NA | NA | NA | yes |
| 6 | Romito 2023 | f | 37 | GNAL exon 3; c.462_463delGA; (p. Lys155Asnfs*9) | - | S | x | x | x | x |  |  |  | NA | NA | NA | NA | yes |
| 7 | Fan 2022 | m | 48 | GNAL c.97C > T; (p.Gln33Ter) | - | Fo |  |  | x |  |  |  | x | NA | NA | NA | anxiety, depression, insomnia | no |
| 8 | Erro 2020 | m | 4 | GNAL exon 2; c.401C>T (p.Thr134Ile) | - | G | x |  | x |  | x |  | x | left upper limb bradykinesia | NA | NA | cerebellar syndrome (tandem gait difficulties, hypometric and slowsaccadic eye movements, dysmetria) | no |
| 9 | Kumar 2019 | f | 32 | GNAL c.667G > A (p.Val223Met) | + | S | x |  | x |  | x |  |  | NA | NA | NA | NA | no |
| 10 | Geoghegan 2019 | f | 1 | GNAL exon 2; c.413A>G, (p.Gln138Arg) | + | G |  |  | x |  | x |  | x | no | NA | NA | myoclonus | no |
| 11 | Ahn 2019 | m | 12 | GNAL exon 12, c.1060G>A; (p. Val354Met) | - | G |  |  |  | x | x | x |  | video right dominant bradykinesia | NA | 200 mg/day, no effect | NA | yes |
| 12 | Pandey 2019 | f | 47 | GNAL exon 12, c.1286G>A; (p.Cys429Tyr) | - | G |  |  | x | x |  | x |  | camptocormia | NA | 400mg/d, minimal response (reduction of camptocormia) | NA | yes |
| 13 | Sarva 2019 | f | 38 | GNAL T197fsX09 | NA | S |  |  | x |  | x |  | x | NA | NA | NA | NA | yes |
| 14 | Sarva 2019 | m | 42 | GNAL R21X | NA | Fo | x |  | x |  |  |  |  | NA | NA | NA | NA | yes |
| 15 | Sarva 2019 | f | 18 | GNAL S95fsX09 | NA | G | x | x |  |  | x | x |  | NA | NA | NA | NA | yes |
| 16 | O'Connor 2017 | m | <20 | GNAL exon 2; c401C>T; (pThr134IIE) | - | G | x | x | x | x | x | x |  | camptocormia | NA | NA | myoclonus | no |
| 17 | Dos Santos 2016 | f | 23 | GNAL c.399C>A (p.F133L) | - | Fo | x | x | x |  |  |  |  | no | NA | NA | NA | no |
| 18 | Carrechio 2015 | m | 36 | GNAL exon 9 c.628G>A; (p.Asp210Asn) | + | Fo |  |  | x |  |  |  | x | slight upper limb bradykinesia | NA | Yes, no effect | NA | yes |
| 19 | Carrechio 2015 | f | 42 | GNAL exon 9 c.628G>A; (p.Asp210Asn) | + | S | x | x | x |  | x |  | x | slight upper limb bradykinesia | NA | NA | myoclonus | yes |
| 20 | Carrechio 2015 | m | 58 | GNAL exon 9 c.628G>A; (p.Asp210Asn) | + | Fo |  |  |  |  | x |  | x | resting tremor, upper limb bradykinesia | yes | yes, no effect | CIDP, posttraumatic hemorrhage without neurological sequelae, gliosis left caudate | no |
| 21 | Kumar 2014 | m | 40 | GNAL exon 8; c.637G>A; (p.Gly213Ser) | - | Fo | x |  | x |  |  |  | x | NA | NA | NA | NA | no |
| 22 | Dobrici 2014 | f | 40 | GNAL exon 12, c.1061T>C (p.Val354Ala) | - | Fo |  |  | x |  |  |  |  | NA | NA | NA | anxiety | no |
| 23 | Ziegan 2014 | f | 54 | GNAL exon 4, c.289A>G (p.Met97Val) | - | S |  |  | x | x |  |  |  | slight upper limb bradykinesia (video, less pronounced post DBS) | NA | NA | depression | yes |
| 24 | Ziegan 2014 | f | 35 | GNAL exon 2, c.166_167insA (p.Ser56-Lysfs*16) | - | Fo |  |  | x |  |  |  |  | NA | NA | yes, no effect | NA | no |
| 25 | Braccia 2024 | f | 33 | 18p- (deletion of 14.19–14.22 Mb in the 18p11.32p11.21region: arr[hg19] 18p11.32p11.21 (14275_14206245x1,14220801x2)) | NA | G | x |  | x |  | x | x | x | no | NA | NA | short stature, short webbed neck, microcephaly, and bilateral eyelid ptosis, hallucinations, severe intellectual disability | no |
| 26 | Braccia 2024 | m | 40 | 18p- (deletion of 13.33–13.37 Mb in the 18p11.32p11.21region: arr[hg19] 18p11.32p11.2 (118729x2, 118788_1346377x1,13496784x2)) | NA | G | x |  | x |  | x | x |  | hypokinesia | yes | yes, moderate benefit for limb hypokinesia/clumsiness | short stature, craniofacial dysmorphism, ptosis, moderate intellectual disability, autoimmune thyroiditis, and pernicious anemia | no |
| 27 | Rauso 2024 | m | 55 | 18p- (hg19, chr18: 10633666-11904117) | - | MF |  |  |  |  | x | x |  | hemiparkinsonism, camptocormia, gait disorder | yes | 300mg/d, levodopa challenge off-med UPDRS III= 25 points vs. on-med UPDRS III= 19 points; improvement of 24%), no benefit for dystonia | NA | no |
| 28 | Vela Desojo 2019 | f | 20 | 18p- (46,XX, [p11]18p) | - | G | x |  | x | x | x |  |  | no | NA | Yes, no effect | dysmorphic features such as dysplastic and low‐set ears, low capillary line, and pterigium colli | yes |
| 29 | Vela Desojo 2019 | f | 40 | 18p- (46,XX, t[18;22][q11;p11]18p-) | - | S |  |  | x |  | x |  |  | upper limb bradykinesia | NA | NA | myoclonus, short stature and facial dysmorphic features such as dysplastic ears, microcephaly, ptosis, and hypertelorism | no |
| 30 | Duma 2019 | f | 26 | 18p- syndrome | - | G | x | x | x |  | x |  |  | no | NA | NA | short stature, dysmorphic facies, restricted vertical gaze, minimal fragmented speech, pituitary abnormalities (hyperprolactinemia, growth hormone deficiency), autoimmune hepatitis, mediastinal masses | yes |
| 31 | Crosiers 2018 (*) | m | 12 | 18p- (deletion of 18p11.32p11.21 (14.39 Mb)) | NA | G | x |  | x | x | x | x |  | no | no | no | short stature, bilateral mild ptosis, divergent strabismus, and a round face with short philtrum, intellectual disability | no |
| 32 | Kumar 2017* | f | 38 | 18p- (14,520-kB loss of 18p11.32) | - | MF | x |  |  |  |  | x |  | NA | no | 300mg/d, improved lower limb dystonia and gait after 6 months | appendicular ataxia (vertical supranuclear gaze palsy, but no gait ataxia), pyramidal tract signs, facial grimace | no |
| 33 | Esposito 2014* | f | 19 | 18p- syndrome | - | MF |  |  | x |  | x | x |  | FOG | NA | Yes, no effect | myoclonus, short stature, round face, bilateral ptosis and microphthalmia, speech and motor development delay | no |
| 34 | Kowarik 2011* | f | 28 | 18p- (46,XX, 18p-) | - | Fo | x |  |  | x | x | x |  | NA | NA | Yes, no effect | Myoclonus, blepharospasm | no |
| 35 | Postma 2009* | f | 30 | 18p- (45,XX,−18,−21,+der(18)t(18;21)(p11;q11)) | - | Fo |  |  |  | x |  | x |  | NA | NA | Yes, no effect | tremor, myoclonus | no |
| 36 | Graziadio 2009* | f | childhood | 18p- syndrome | NA | MF/G | x |  | x | x |  |  |  |  | NA | NA | blepharospasm | no |
| 37 | Nasir 2006* | f | adolescence | 18p- syndrome and a chromosomal rearrangement involving chromosome 14 | + | MF |  | x | x |  | x |  |  | global rigidity | NA | NA | vocal tics, blepharospasm | no |
| 38 | Klein 1999* | f | 12 | 18p- syndrome | NA | S |  | x | x | x |  |  |  | NA | NA | NA | blepharospasm | no |
| 39 | Klein 1999* | f | 15 | 18p- syndrome | NA | S/G |  |  |  | x | x | X |  | NA | NA | NA | NA | no |
| 40 | Klein 1999* | m | 17 | 18p- syndrome | NA | MF |  | x | x | x |  | x |  | NA | NA | NA | blepharospasm | no |
| 41 | Awaad 1999* | f | <20 | 18p- (karyotype 46, XY, del (18)(p11.1)) | - | G |  |  | x | x | x | x |  | NA | NA | Yes, no effect | Chorea, oromotor apraxia, | no |
| 42 | Tezzon 1998* | m | NA | 18p- syndrome | NA | G | NA |  |  |  |  |  |  | hypokinesia | NA | NA | NA | no |
| 43 | Kakinuma 1994* | NA | NA | 18p- syndrome | NA | Fo | NA |  |  |  |  |  |  |  | NA | NA | Pyramidal tract signs | no |

**ESM 5** Demographical, genetical and clinical overview of all presented and reviewed DYT-GNAL and 18pdel-dystonia patients with an overview of affected body region at longest follow-up and bradykinetic symptoms, Dopamine transporter (DaT) scan results and levodopa response if applicable as well as additional features and use of deep brain stimulation (DBS). Publications indicated with * are implemented in the review written by Crosier et. al. 2018. **Abbreviations**: f=female, m=male; NA=not available, AAO=age at onset, (+)=positive, (-)=negative, Fo=focal, MF=multifocal, S=segmental, G=generalized; Site: F=face, L=larynx, C=cervical, T=trunk, UL=upper limb, LL=lower limb, T=tremor
