# Supplementary material for: Long-term efficiency of pallidal DBS and the role of Levodopa treatment in DYT-GNAL and 18p deletion syndrome associated dystonia: an observational study and review of literature
Source: J Neural Transm (Vienna). 2026 Feb 3;133(9):2907–16. doi: 10.1007/s00702-026-03109-z (PMC13687376; doi:10.1007/s00702-026-03109-z)
Supplement: Supplementary file 2 [file 702_2026_3109_MOESM5_ESM.docx]

**Supplementary Information (SI) – ESM 5**

**Long-term efficiency of pallidal DBS and the role of Levodopa treatment in DYT-GNAL and 18p deletion syndrome associated dystonia: An observational study and review of literature**

**Journal of Neural Transmission**

**Authors:** Reimer, Johanna^1,2^; Schumann, Friederike^1,3^; Lohmann, Katja^4^; Riemer, Thomas G.^5^; Krauss, Joachim K.^6^; Schneider Gerd-Helge^7^; Kühn, Andrea A.^1,8^; Krause, Patricia^1^

1 Department of Neurology, Movement Disorder and Neuromodulation Unit, Charité-Universitätsmedizin Berlin, Berlin, Germany

2 Berlin Institute of Health, Charité-Universitätsmedizin Berlin, Berlin, Germany

3 Department of Neurology, Park-Klinik Weißensee, Berlin, Germany

4 Institute of Neurogenetics, University of Lübeck, German

5 Department of Psychiatry and Psychotherapy, Charité-Universitätsmedizin Berlin, Berlin, Germany; Institute of Clinical Pharmacology and Toxicology, Charité-Universitätsmedizin Berlin, Berlin, Germany

6 Department of Neurosurgery, Hannover Medical School, Hannover, Germany.

7 Department of Neurosurgery, Charité-Universitätsmedizin Berlin, Berlin, Germany

8 NeuroCure Cluster of Excellence, Charité-Universitätsmedizin Berlin, Berlin, Germany

**Corresponding author**: Patricia Krause, MD, patricia.krause@charite.de; ORCID 0000-0001-9063-0556

| **ID** | **Sex** | **AAO** | **Age DBS** | **FU** | **Symptoms** | **Fo/S/G** | **Target** | **Settings** | **Results** |
| --- | --- | --- | --- | --- | --- | --- | --- | --- | --- |
| 1 | m | 36 | 44 | 11 | CD, upper and lower face dystonia, right upper limb | G | GPi | L: 2.6V, 210Hz, 60µs; R: 3.8V, 210Hz, 60µs | Immediate response 80-90% symptom relief (surgery in MSH Hannover). secondary generalization to upper and lower limbs under DBS. Sustained sufficient relief of cervical dystonia and back pain. Side effects: dysarthria |
| 2 | f | 31 | 34 | 25 | phasic CD, dystonic tremor, trunk, right upper and lower limb dystonia, pain | G | GPi (initially + VIM) | L: 1,7 mA, 185Hz, 90µs; R: 1,9 mA, 185Hz, 90µs | complete relief of torticollis, truncal and limb dystonia, suppression of phasic movement, reduced pain (TWSTRS II at late FU 0), side effects mild dysphagia and slowness of speaking, explantation of VIM DBS due to infection, no decline of treatment success with GPi DBS alone |
| 3 | m | 28 | 35 | 7 | phasic CD with dystonic head tremor, pain | S | GPi | L: 9,10-, 2mA, 179Hz, 120µs;  R: 1,2-, 2.9mA, 179Hz; 120µs | Complete relief after 1 year lasting up to 7 years of follow up, initially no effect with standard Settings (130Hz 90µs); dystonic upper limb tremor in disease course, complete pain relief, TWSTRS II from 7 at baseline to 0 at early and late FU) |
| 5 | f | 7 | 39 | 1 | Blepharospasm, cervical dystonia with writer’s cramp, action tremor and dystonic body posture | S | GPi | NA | significant reduction in the BFMDRS from 40 preoperative (31 movement scale, 9 disability scale) to 10 (8 movement scale, 2 disability scale) 6 months after the procedure. Best responding: blepharospasm, cervical dystonia, posture |
| 6 | f | 37 | 42 | 7 | CD, truncal, and laryngeal dystonia | S | GPi | L: C+1-2-, 3.3V, 120μs, 180 Hz,  R: C+9-10-, 3.0 V, 120 μs, 180 Hz | Significant improvement in axial and cranial district dystonia, speech included. Dysarthria persisted partially |
| 11 | m | 12 | 19 | 2 | Trunk, upper extremities | G | GPi | L: C+2-, 2.5V, 150μs, 150Hz;  R: C+2-, 2.5V, 150μs, 150Hz | 70% improvement of truncal and hand dystonia |
| 12 | f | 47 | N/A | 1 | CD, trunk, lower extremity dystonia | G | GPi | NA | Significant improvement of cervical, truncal, and leg dystonia |
| 13 | f | 38 | 53 | 2 | CD and upper limb dystonia, head tremor | S | GPi | L: 0-3+, 4V, 90μs, 185Hz; R: 8-11+, 6V, 120μs, 185Hz | 85% improvement of neck and upper limb dystonia, after 1year. pain improved, after 2 years arm tremor reduced, mild residual laterocollis, writer’s cramp persisted |
| 14 | m | 42 | 53 | 8 | Cervical dystonia | Fo | GPi | L: C+ IL 0/1-, 3V, 90μs, 125Hz;  R: C+ IL 1/2-, 3V, 90μs, 125Hz | 50% improvement of neck dystonia, 3 years after DBS dystonic posturing improved further, pain and dysphagia persisted |
| 15 | f | 18 | 41 | 8 | Cranial (pharynx, upper and lower face, tongue, jaw), both arms and legs | G | GPi | L: C+1-, 4.5V, 60μs, 145Hz;  R: C+5-, 4.2V, 90μs, 145Hz | 85% improvement of neck and 90% improvement of foot dystonia, progression and persistence of dysarthria and left-hand dystonia |
| 18 | m | 36 | 55 | 4 | CD, head tremor | Fo | GPi | L: C+1-, 2.5V, 90μs, 130Hz;  R: - 8+9, 2V, 90μs, 130Hz | Substantial clinical subjective and objective improvement, subjectively 80-90% symptom reduction |
| 19 | f | 42 | 62 | <1 | Head and limbs tremor, CD and laryngeal dystonia | S | GPi | L: C+ IL 0 (1.25V)/  1 (2V)-, 60μs, 125Hz;  R: C+ IL 1 (2.15V)/ 2(2V)-, 60μs, 125Hz | immediate improvement of cervical and laryngeal dystonia |
| 23 | f | 54 | 60 | 5 | CD and truncal dystonia | S | GPi | L: C+8-, 2.3V, 120μs, 180Hz;  R: C+0-, 2.3V, 120μs, 180Hz | 80% BFMDRS improvement especially in truncal dystonia |
| 28 | f | 20 | 24 | 1 | CD, neck pain, facial grimacing. spreading to upper limbs and trunk | G | GPi | NA | improvement of the BFMDRS parts I and II were 98.4% and 100% |
| 30 | f | 26 | 33 | 1 | CD, Laryngeal, right upper limb, painful spasms, progression to generalization | G | GPi | L: 3.5 V 90 µs, 130 Hz; R: 3.2 V, 90 µs, 130 Hz | functional independence, being able to mobilize, feed herself, and communicate. Mild dystonia persisted, particularly involving the face and arms (pre op bedridden and mute due to laryngeal dystonia) |

**ESM 6** Demographical and clinical overview of all DYT-GNAL and 18pdel-dystonia patients with DBS including individual deep brain stimulation (DBS) settings and results according to affected body region at longest follow-up. **Abbreviations**: f=female, m=male; AAO=age at onset; CD=cervical dystonia; Fo=focal, MF=multifocal, S=segmental, G=generalized; DBS=deep brain stimulation; GPi=internal Globus pallidus, VIM=ventral intermediate nucleus of the thalamus, NA=not available, L=left, R=right, C=case, IL=Interleaving, TWSTRS=Toronto Western Spasmodic Torticollis Rating Scale I (Severity scale), II (Disability scale), III (Pain Scale); BFMDRS=Burke-Fahn-Marsden Dystonia Rating Scale I (Dystonia movement scale) and II (disability scale)
